# Supplementary material for: Characterization and Treatment Responsiveness of Genetically Engineered Ornithine Transcarbamylase-Deficient Pig
Source: J Clin Med. 2021 Jul 22;10(15):3226. doi: 10.3390/jcm10153226 (PMC8347267; doi:10.3390/jcm10153226)
Supplement: Supplementary file 1 [file jcm-10-03226-s001.zip › jcm-1288608-SI.pdf]

Supplementary Materials

Table S1. Pregnancy and delivery outcomes for ornithine transcarbamylase deficient (OTCD) carrier female pigs.

**Mother pig ID** [1]	**K76**	**K77**	**K77(2)**	**K78(2)**	**K79**	**K80**	**K96**	**K96(2)**	**K96(3)**	**K97**	**K100**	**K103**	**K103(2)**	**K106**	**K106(2)**	**K135**	**W321**	**W328**	**Total No.**
Gestation length, day	112	112	111	111	114	112	106	109	111	106	112	110	111	112	111	112	114	112	
Divergence [2]	-2	-2	-3	-3	0	-2	-8	-5	-3	-8	-2	-4	-5	-2	-3	-2	0	-2	
Mode of delivery [3]	N	N	N	H	N	N	N	N	H	H	N	N	N	N	H	O	O	H	
Total No. of piglets	13	7	8	11	7	11	12	9	13	10	8	11	6	11	11	7	4	14	173
No. of live piglets	9	7	8	10	5	11	9	8	12	10	8	10	6	10	10	7	4	9	153
**Live, male, OTCD** [4]	**2**	**1**	**1**	**0**	**0**	**1**	**1**	**2**	**1**	**2**	**1**	**2**	**1**	**0**	**1**	**2**	**1**	**2**	**21**
Live, Male, WT [5]	4	2	3	2	1	3	1	2	1	4	1	6	1	3	1	2	1	2	39
Live, female, carrier	0	2	2	3	4	3	3	2	6	4	5	1	1	2	1	3	2	2	46
Live, female, WT	3	2	3	5	0	4	4	2	4	0	1	1	3	5	3	1	0	2	42
No. of dead piglets	4	0	0	1	2	0	3	1	1	0	0	1	0	1	1	0	0	5	20
**Dead, male, OTCD**	**4**	**0**	**0**	**1**	**2**	**0**	**3**	**1**	**1**	**0**	**0**	**1**	**0**	**0**	**1**	**0**	**0**	**5**	**19**
Dead, male, WT	0	0	0	0	0	0	0	0	0	0	0	0	0	1	0	0	0	0	1
Dead, female, carrier	0	0	0	0	0	0	0	0	0	0	0	0	0	0	0	0	0	0	0
Dead, female, WT	0	0	0	0	0	0	0	0	0	0	0	0	0	0	0	0	0	0	0

[1] Number in parenthesis denotes the number of delivery times; [2] Divergence from standard gestation period (114 days [7]); [3] N; normal delivery, H; hysterotomy after the sign of delivery, O; with oxytocic treatment; [4] OTCD; Ornithine transcarbamylase deficient; [5] WT; wild type; [6] Average gestation length of total cases (n=18) and cases of normal delivery (n=11) were both 111 days.
